# Nanoencapsulation of morin hydrate with BSA for sustained drug release in colorectal carcinoma cells: experimental and computational approach

**DOI:** 10.3389/fddev.2025.1623317

**Published:** 2025-08-13

**Authors:** Sanju Kumari Singh, Amit Kumar Srivastav, Sunaina Chaurasiya, Sunita Patel, Umesh Kumar, Hitesh Kulhari

**Affiliations:** ^1^School of Life Sciences, Central University of Gujarat, Gandhinagar, Gujarat, India; ^2^School of Nano sciences, Central University of Gujarat, Gandhinagar, Gujarat, India

**Keywords:** colorectal cancer, bioavailability, nanoparticles, crystalline, amorphous

## Abstract

Colorectal cancer is among the most redundant cancer of the gastrointestinal tract, with its burden expected to rise 60% by 2030. Morin hydrate (MH) is a bioflavonoid with anticancer attributes. However, the implementation of MH is limited due to its hydrophobic properties, along with poor stability and bioavailability. Protein-based nanoparticle may encapsulate the drug and this complex can enhance the drug efficacy and delivery to colorectal carcinoma cells. To investigate the molecular interactions between BSA and MH, the Lamarckian genetic approach was used. In the current study, we prepared BSA encapsulated MH nanoparticles by desolvation method. The characterization of the nanoparticles was done by XRD, DSC, TGA and FTIR was performed to corroborate the results. MHNPs were spherical with a particle size of 90 nm determined by TEM and a zeta potential of −11 ± 5.90 mV. BSA nanoparticles improve the thermal stability and sustained release profile of Morin Hydrate, enabling its application as a phytochemical-based anticancer nanocarrier. The antioxidant test of MHNPs showed higher radical scavenging ability than MH. Additionally, our release investigations show that drug release occurs from the matrix of the nanoformulation to reach the target site efficiently. An increase in the anticancer potential was shown by an *in vitro* cytotoxicity assay in comparison to MH. These data suggest that MH was successfully encapsulated and enhanced solubility, resulting in greater bioavailability.

## 1 Introduction

Herbal traditional medicines are being extensively used for the treatment of various ailments from the medieval periods. Morin hydrate (MH) is a naturally occurring flavonoid with distinctive biological characteristics, a pentahydroxy flavone found in the leaves, stems, and fruits of numerous plants, including figs from the moraceae family (Venu Gopal, 2013). This is a flavanol with anti-inflammatory (Hsieh et al., 2023; Touny et al., 2024), antioxidant (Famurewa et al., 2023), and free radical scavenging characteristics, which assist in the treatment of several disorders. MH shows numerous pharmaceutical applications and its benefits have been revealed in a number of recent research. MH has anti-cancer properties against several malignancies (Chandra et al., 2023; Balaga et al., 2023; Goyal et al., 2023). Furthermore, it possesses potential neuroprotective, nephroprotective, hepatoprotective, gastroprotective, and cardioprotective abilities (Singh et al., 2013). Furthermore, in light of the aforementioned findings, MH has the potential to be employed as a new medicine; however, in this scenario, its healing efficiency and side effects should be evaluated through clinical trials. So, the preventive benefits of MH can also be employed as a medicinal cure by enough scientific backing (Balaga et al., 2023; Singh and Patel, 2024). Cancer is one of the deadliest diseases causing major mortality globally. Colorectal cancer (CRC) is the third most often reported cancer globally. Colon is a lengthy tube that helps carry digested food to the rectum and subsequently out of the body. This is the first site where colorectal cancer appears initially (Sok et al., 2021). Colorectal cancer (CRC) is a leading cause of cancer-related mortality worldwide, distinguished by its complex pathology and resistance to conventional therapies (Shen et al., 2024) (Sheik and Huh, 2022). Phytochemicals like morin hydrate have emerged as potential therapeutic agents due to their anticancer properties. However, their clinical application is often limited by poor solubility and bioavailability. Nanoparticle drug delivery systems, such as those encapsulating morin hydrate and bovine serum albumin (BSA), offer innovative solutions to enhance drug efficacy and targeting in CRC treatment. The following sections will explore the significance of CRC, the role of morin hydrate, and the advantages of BSA-encapsulated nanoparticles in drug delivery. If CRC is not recognized early and treated, then it might migrate to saveral organs or regions in the body. Clinical studies indicate that drugs with low solubility absorb poorly in the colon, which may be exacerbated by the colon’s low availability of free fluid (McCoubrey et al., 2023; Tannergren et al., 2009). Providing precise drugs in a tailored to the site and focused on targets approach can enhance chronic disease therapy in a variety of ways (Patel et al., 2020a). In recent years, there have been significant developments in nanomedicine for the treatment of several illness (Elsayed et al., 2021), including CRC. Pharmaceutical delivery vehicles are one of the various drug packaging solutions that allow the drug to enter the body safely (Solanki et al., 2021a). Liposomes, nanoparticles, and micelles are commonly used as drug delivery vehicles (Kumari Singh et al., 2024) (Patel et al., 2020b).

## 2 Materials and methods

### 2.1 Materials

Bovine serum albumin (BSA) (98%, 66 kDa), Morin hydrate, ethanol, Ethidium Bromide (EtBr), glutaraldehyde, 3-(4,5-dimethylthiazol-2-yl)-2,5-diphenyl tetrazolium bromide (MTT), Dimethyl Sulphoxide (DMSO) and Acridine Orange (AO) were procured from Sigma Aldrich (Saint Louis, MO, United States). 0.25% Trypsin EDTA, Fetal Bovine Serum (FBS) and Roswell Park Memorial Institute (RPMI) were obtained from thermofischer scientific (Waltham, MA, United States). HCT 116 cell line was procured from cell repository at CIF, CUG. DPPH (2,2-Diphenyl-1-picrylhydrazyl) (0.1 mM) was sourced from TCI (Tokyo Japan), while methanol from (Loba Chemie) Mumbai, India.

### 2.2 Synthesis of BSA encapsulated morin hydrate nanoparticles (MHNP)

**FIGURE 1:**
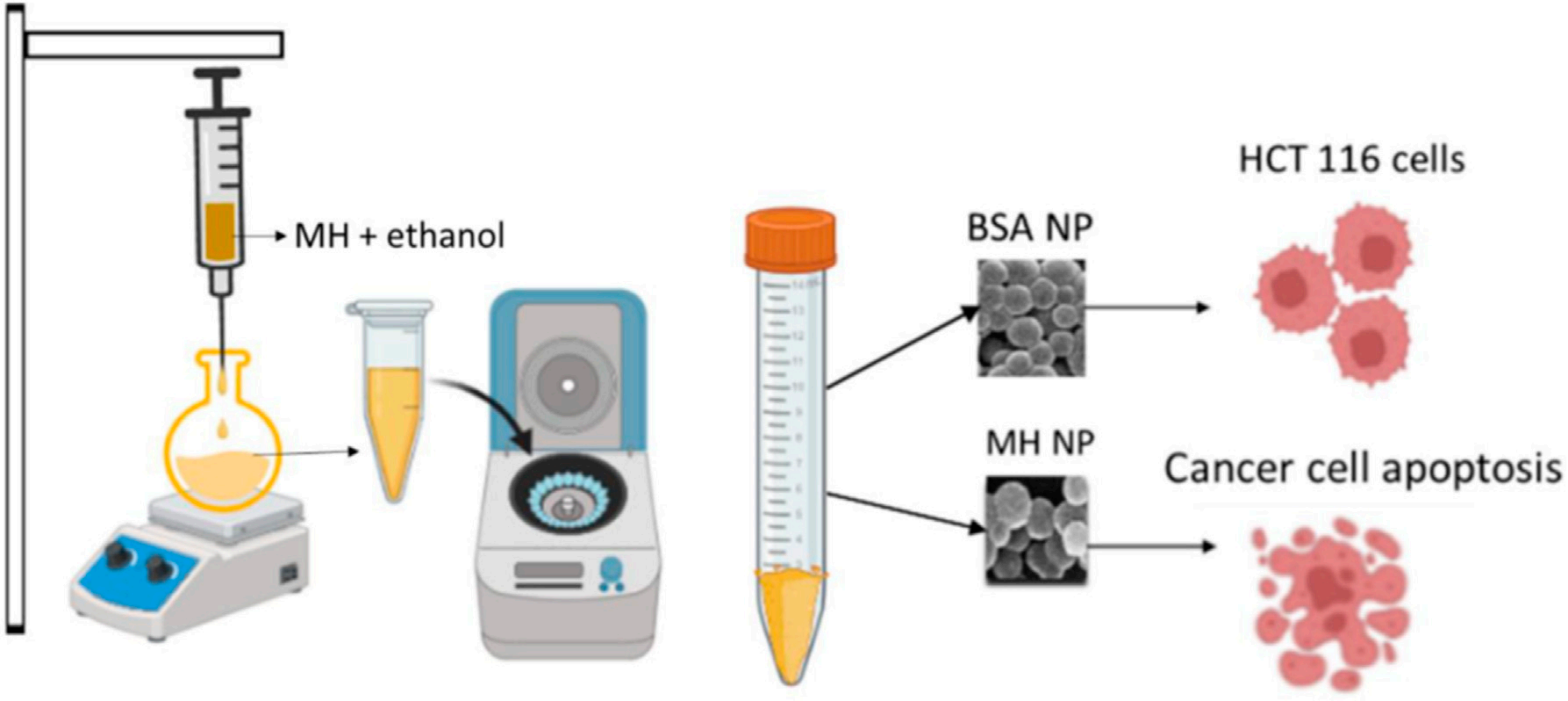
Schematic representation of synthesis of MHNPs.

BSA nanoparticles and BSA encapsulated MH nanoparticles were prepared by desolvation method (Tanjung et al., 2024). After few reactions for optimization of drug encapsulation and release patterns, 100 mg BSA, 20 mg of MH with a crosslinker volume of 150 μL at pH 9 with a stirring speed of 550 rpm was finalized. Firstly, BSA was dissolved in distilled water. pH of the solution was set to the required value. Reaction was set at 500 rpm. Subsequently, 8 mL of MH in ethanol solution was added to the reaction vial with a flow rate of 1 mL/min. Glutaraldehyde, the crosslinker for the reaction was also added to the reaction vial with same flow rate. After 22 h, the reaction was stopped and the prepared nanoparticles were purified by four cycles of centrifugation at 12,500 rpm for 15 min at RT and 4 cycles of sonication for 10 min in between every centrifugation. Figure 1 shows a diagramatic diagram of preparation of nanoparticles. To confirm the encapsulation of drug with BSA, UV was done using Evolution 201 UV-Visible spectrophotometer (Thermoscientific). The optical characteristics were measured in 10-mm optical path-length cuvettes with wavelengths ranging from 200 to 600 nm. Equivalent volumes of the suspension (0.5 mL) were diluted in a consistent volume of ultrapure deionized water (Milli Q) (2 mL), and then assessed (Hanafy et al., 2023).

### 2.3 Characterization of BSANPs and MHNPs

#### 2.3.1 PSA (particle size analyzer) and zeta potential measurement

Separately, 500 mg of BSANPs and MHNPs were dissolved in distilled water, of the resulting aqueous solution were used to determine the particle size (Solanki et al., 2024a). A hydrodynamic size assessment was conducted for the BSANPs and MHNPs by the particle size analyzer (Malvern Zetasizer Nano-S90). Moreover, zeta potential of the formulation was also determined.

#### 2.3.2 FE-SEM (field Emission scanning electron Microscopy)

Surface morphology of prepared MHNPs was observed using FE-SEM (JEOL, JSM7600F, Japan). A known amount of BSANPs and MHNPs was dissolved (Solanki et al., 2021b) 10 µL of the solution was placed onto silicon wafers dried and observed under a voltage of 10 kV. A frequency distribution curve was plotted using ImageJ.

#### 2.3.3 FTIR (Fourier transform infrared spectroscopy)

The encapsulation of MH in BSA was confirmed by FTIR analysis, known quantity of pure MH, pure BSA, BSANP and MHNPs was subjected to analysis using FTIR (Nicolet iS5, Thermo Scientific, United States) in the range of 4,000 to 500 cm^-1^.

#### 2.3.4 XRD (X-ray diffraction)

The physical state of prepared nanoparticles was confirmed by XRD (Aldeen et al., 2022). For that, XRD patterns of pure MH, pure BSA, BSANP and MHNPs was recorded using X-ray diffractometer (Analytical, Almelo, Netherlands) at a voltage of 40 kV, the operating current was 30 mA and 2θ angle ranging from 5° and the finishing angle was 50°.

#### 2.3.5 DSC (differential scanning calorimetry)

MHNPs’ thermodynamic characteristics were assessed using DSC (DSC 6000, PerkinElmer, United States) (Wesolowski and Leyk, 2023). Samples were placed within conventional aluminum pans and subjected to scanning under nitrogen conditions at a flow rate of 20 mL per minute, while the temperature range was monitored between 30°C and 300°C. An empty aluminum pan was utilized as a point of reference (Solanki et al., 2024a).

#### 2.3.6 TGA (thermogravimetric analysis)

TGA was done to assess the nanoparticles’ thermal stability (Loganathan et al., 2017). A known amount of pure MH, pure BSA, pure BSANPs, and MHNPs were taken and subjected to TGA analysis (TG/DTA 7300, exstar). Thermograms in the temperature range of 30 °C–700 °C were measured at a heating rate of 10°C min−1.

#### 2.3.7 Stability studies

The stability of nanoparticles was monitored for a duration of 30 days. PDI (Polydispersity index) measures how homogenous or heterogenous a sample is (Rohiwal et al., 2015) in terms of size. The PDI, size and solubility of the nanoparticles at pH 5 and pH 7.4 was determined.

### 2.4 Molecular docking (MD)

MD simulations were performed to characterize the binding affinity and potential interactions between bovine serum albumin (BSA) and MH. The AutoDock v4.2 software package was employed, utilizing the Lamarckian genetic algorithm to conduct a comprehensive exploration of the conformational space (Morris et al., 2009; Eberhardt et al., 2021). MH, a compound recognized for its biological activities, served as the ligand in this study. The receptor protein, BSA, was retrieved from the Protein Data Bank (PDB) and subjected to rigorous preprocessing to remove extraneous molecules and ions (Bujacz, 2012). The ligand structure was optimized and converted into a suitable format prior to docking. Critical docking parameters, including the definition of the search space and scoring function, were meticulously determined. The resultant docking poses were subjected to a thorough analysis to identify optimal binding conformations. Visualization and in-depth analysis of the docked complexes were performed using specialized molecular graphics software, i.e., Pymol, Chimera, VMD (Pettersen et al., 2004; Humphrey et al., 1996; Lineback and Jansma, 2019).

### 2.5 MD simulation

MD simulations were employed to investigate the dynamic and static behavior of the BSA-MH complex within a simulated aqueous environment. The GROMACS v2022 software package was used to conduct these simulations (Abraham et al., 2015). The BSA-MH complex, generated from the docking studies, was embedded in a periodic cubic box filled with explicit water molecules. The TIP3P water model was employed for its computational efficiency and reasonable accuracy in representing water properties (Mark and Nilsson, 2001). The net charge of the system was neutralized by adding counterions. The CHARMM36m force field was utilized to describe the bonded and non-bonded interactions within the BSA, MH, and water molecules (Huang et al., 2017). Default parameters were applied for BSA-MH MD simulation studies (Srivastav et al., 2020). To mitigate potential artifacts arising from the initial system configuration, an equilibration phase was implemented. Energy minimization to remove steric hindrance was done (Mackay et al., 1989). Following the initial setup, the system was subjected to position restraints on the heavy atoms of both the protein and the ligand during an NVT ensemble simulation. This step was essential to gradually bring the system’s temperature to the target level. Subsequently, an NPT ensemble simulation was conducted, allowing the system to equilibrate under the desired pressure conditions. After achieving equilibrium, a production MD simulation was run for a duration of 100 nanoseconds. All positional constraints were lifted during this phase, allowing the system to evolve organically under recurring boundary conditions. The Nose-Hoover thermostat was used to maintain the temperature at 300 K and the pressure of 1 atm (Sidler and Riniker, 2019). To guarantee accuracy, Long-range electrostatic interactions were estimated using the Particle Mesh Ewald (PME) method (Srivastav et al., 2024; Simmonett and Brooks, 2021). To derive meaningful insights from the generated trajectory, extensive analysis was performed. The binding free energy of the ligand and protein was evaluated using the Molecular Mechanics Generalized Born Surface Area (MM/GBSA) method, which provided an evaluation of the interaction strength (Genheden and Ryde, 2015). Additionally, the dynamic behavior of the ligand within the binding site was examined to identify key residues that play a crucial role in ligand recognition and binding.

### 2.6 in vitro drug release study

The drug release rate of nanoparticles was tested at 37°C across three different pH levels: 7.4, 6.8, and 5.5. The pH 7.4 medium reflects physiological conditions, while pH 6.8 mimics the intestinal pH, and pH 5.5 corresponds to the acidic conditions found in the mature endosomes within tumor cells (Scialla et al., 2023; Jangid et al., 2022). The release profiles of MH from MHNPs were studied using a dialysis bag (MWCO-6000). Briefly, MHNPs were disseminated in PBS buffer in a dialysis bag. It was submerged in 150 mL of buffer solution (pH 7.4, 6.8, or 5.5) before being put on a magnetic stirrer set to 100 rpm at 25 °C. At predefined intervals, 2 mL of the release medium was taken to determine the released drug concentration, which was subsequently replenished with the same fresh buffer. The absorbance of the release media at 263 nm was measured using an Evolution 201 UV-Visible spectrophotometer. All samples were subjected to three separate experiments at each pH level. To predict the *in vitro* drug release status of the nano drug delivery system a release study was conducted using an appropriate dissolving medium that mimicked relevant physiological conditions. The study included Simulated Gastric Fluid (SGF) at pH 1.2 for 3 h, which is around the typical gastric emptying time, Simulated Intestinal Fluid (SIF) at pH 6.8 for 4 h, and Simulated Colonic Fluid (SCF) for 24 h (Wahlgren et al., 2019; McCoubrey et al., 2023). The sample was continuously stirred at 150 rpm and maintained at 37°C ± 0.5°C. The period of testing in each medium is advised by the US, EU, and international pharmacopeias to replicate the transit durations in the stomach, small intestine, and colon (Smyrlaki et al., 2024). The dialysis technique was employed to study the *in vitro* release of MH from MHNP under simulated gastrointestinal conditions. The absorbance of the collected samples was measured at 263 nm using a UV-visible spectrophotometer. MH concentration was determined by referencing a standard curve of the pure drug in the corresponding buffer.

### 2.7 DPPH (2,2-diphenyl-1-picrylhydrazyl) assay for antioxidant study

The DPPH assay is well-known for assessing the antioxidant potential of compounds. A stock solution of DPPH at 1 mM concentration was prepared using methanol, from which a 0.1 mM working stock was subsequently derived for the assay. In a 96-well plate, a series of dilutions from 80 µg/mL-180 μg/mL of pure MH, blank BSA, BSANPs, and MHNPs were added. The final volume was kept to 200 µL with DPPH solution. The DPPH solution alone served as the control. Subsequently, the plate was kept in the dark and incubated for 30 min at RT. The DPPH solution shows a strong absorption band at 517 nm, attributed to its unpaired electron and its dark violet color. Thus, the absorbance was measured at 517 nm using a SYNERGY LX multimode reader (BioTek, United States). Antioxidant inhibition (%) was determined using the formula below (Panhwar and Memon, 2011; Montiel et al., 2024).

$$Antioxidant\;acitivity\left(\%\right)\frac{Absorbance\;of\;control-Absorbance\;of\;sample}{Absorbance\;of\;control}X100$$

### 2.8 cell culture

Anticancer activity of MHNPs was estimated against HCT 116 cells. Cells were maintained in RPMI supplemented with 10% FBS and incubated in a 5% CO_2_ condition at 37°C (Solanki et al., 2023). Cells were monitored routinely and after reaching a confluency around 85%–90%, anticancer studies were performed.

### 2.9 in vitro cytotoxicity study

Firstly, IC50 of MH was determined on HCT116 cells using MTT Assay. 7,500 cells per well were cultured in 96-well plates. After they got adhered and attained their morphology, MH was administered to cells at various concentrations (20, 40, 80, 160, 320, 400, and 440 μg mL^−1^) for 24 h, 48 h, and 72 h. The cells were then treated with MTT for 4 h, and the formazan crystals were dissolved with DMSO (Solanki et al., 2024b). The absorbance at 570 nm was measured using a multimode ELISA plate reader (Synergy H1, United States). Furthermore, the following equation was used to compute the percentage of cell viability.

$$\%\;of\;cell\;viability\frac{O.D\;value\;of\;experimental\;sample}{O.D\;value\;of\;experimental\;control}\;X100$$

The MTT Assay was used to evaluate the cytotoxic effects of MHNPs on HCT116 cells *in vitro*. In 96-well plates, 7.5 × 10^3^ cells were grown. The next day, cells were exposed to varying concentrations of BSANP, MH, and MHNP for 24 and 48 h (80, 100, 120, 140, 160, and 180 μg mL^−1^).

### 2.10 trypan blue dye exclusion assay

Furthermore, cell cytotoxicity was confirmed using the trypan blue assay, a widely used method for determining the number of live and dead cells in a cell suspension (Mani and Swargiary, 2023). The assay is fast and widely reliable. Underlying principle of this assay is cells with cell membranes, inhibit trypan blue dye whereas dead cells allow dye due to their compromised cell membrane, resulting in blue color (Vitipon et al., 2025). 25,000 cells/well were seeded in 24 well plates. After the cells got attached and their morphology was attained by 24 h, cells were treated with different concentrations (80, 100, 120, 140, 160, and 180 μg mL^−1^) of BSANP, MH and MHNPs. Subsequently, the cells were trypsinized using EDTA-Trypsin. The resulting cell suspension was centrifuged at 950 rpm for 10 min at room temperature. After discarding the supernatant, the cells were resuspended in 20 μL of PBS. An equal volume of Trypan blue dye was then added, and the mixture was incubated for 2 minutes. Finally, the cells were counted using a hemocytometer under the phase contrast microscope. Similarly, images were captured 24- and 48-h post treatment.

### 2.11 Apoptosis assay

To evaluate apoptosis, 25,000 HCT116 cells were seeded in 24-well plates. After 24 h, cells were treated with 80 μg/mL MH, BSA NPs, and MHNPs. After the treatment time, Cells were trypsinized and centrifuged at 950 rpm for 10 minutes (Xu et al., 2025). The pellet was re-suspended in PBS and collected for further analysis. On a clean glass slide, 5 μL of EtBr (1 mg/mL) and 5 μL of Acridine orange (1 mg/mL) were mixed with 10 μL of the cell solution. Cells were viewed, and photos were acquired with a fluorescent microscope.

### 2.12 Colony formation assay

The antiproliferative activity of MHNPs was further assessed by using colony formation assay (Rajendran and Jain, 2017). A total of 900 cells per well were seeded into 6-well plates. Once the cells reached morphology, they were treated with 80 μg/mL MH, BSANPs and MHNPs for 3 days. After incubation, cells were rinsed in PBS, fixed with ethanol: glacial acetic acid (95:5), and stained with 2% crystal violet. The number of colonies was tallied, and photos were taken.

### 2.13 statistical analysis

The experiments were conducted in triplicate. Quantification and graph preparation were performed using origin (2022) and GraphPad Prism 9 software.

## 3 Results and discussion

### 3.1 preparation of BSA encapsulated morin hydrate nanoparticles (MHNP)

BSA encapsulated MH nanoparticles were in the range of 168.6 nm in size with a stable PDI of 0.2 at optimized parameters for reaction conditions mentioned in the methods. The average drug entrapment effectiveness of MHNPs was 71.66 ± 1.5% having a loading capacity of 10 ± 0.5 as calculated by UV analysis.

### 3.2 Characterization of BSANPs and MHNPs

#### 3.2.1 PSA and zeta potential measurement

**FIGURE 2:**
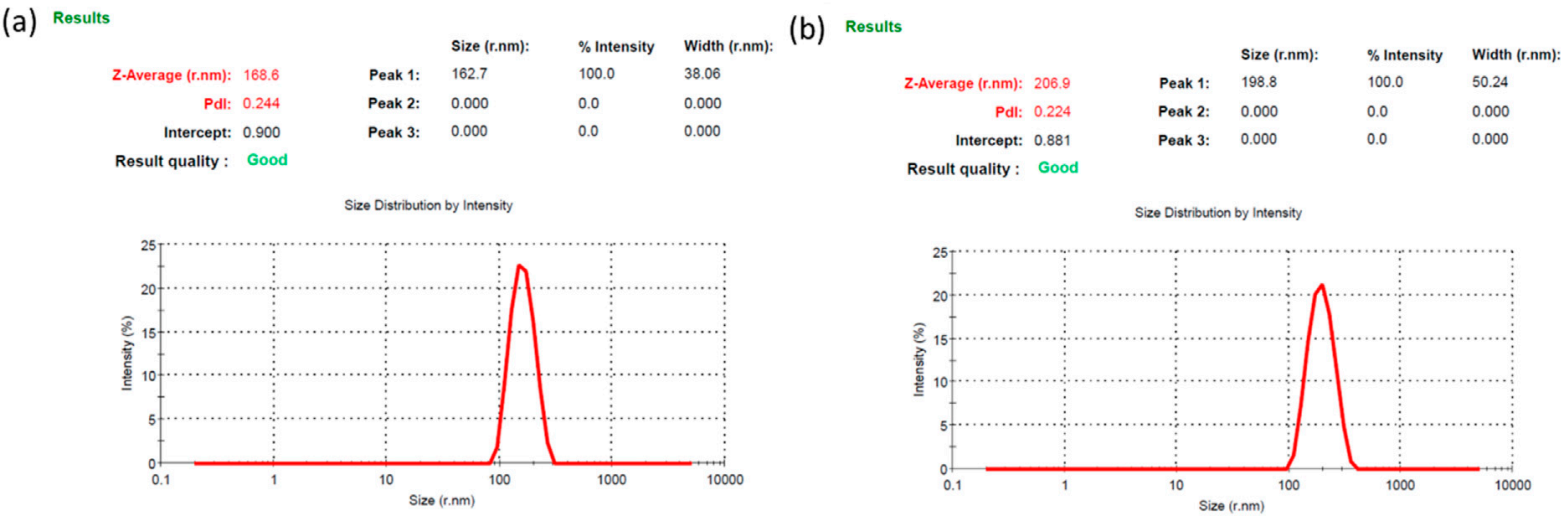
Size of the **(a)** BSANPs and **(b)** MH-NPs determined by PSA (Particle Size Analyzer).

The particle size analyzer data suggested a size of 168.6 nm with PDI 0.244 and 206.9 nm with PDI 0.224 for the BSANPs and MHNPs respectively (Figure 2). The PDI is a dimensionless metric used to express how uniform a distribution is. A PDI up to 0.4 is acceptable, therefore the PDI is within limit. Zeta potential value is considered as a measure of potential aggregation or instability of the particles in dispersed condition. The ζ potentials of BSANPs and MHNPs at pH 7.4 is −7.07 ± 4.51 mV and −11.0 ± 5.90 mV for respectively representing a good stability (Table 1).

**TABLE 1:** Physical attributes of the blank and encapsulated nanoparticles.

Formulation	Size(nm)	PDI	Zeta potential (mv)
BSANP	168.6	0.244	−7.07
MHNP	206.9	0.244	−11

#### 3.2.2 FE-SEM and HR-TEM

**FIGURE 3:**
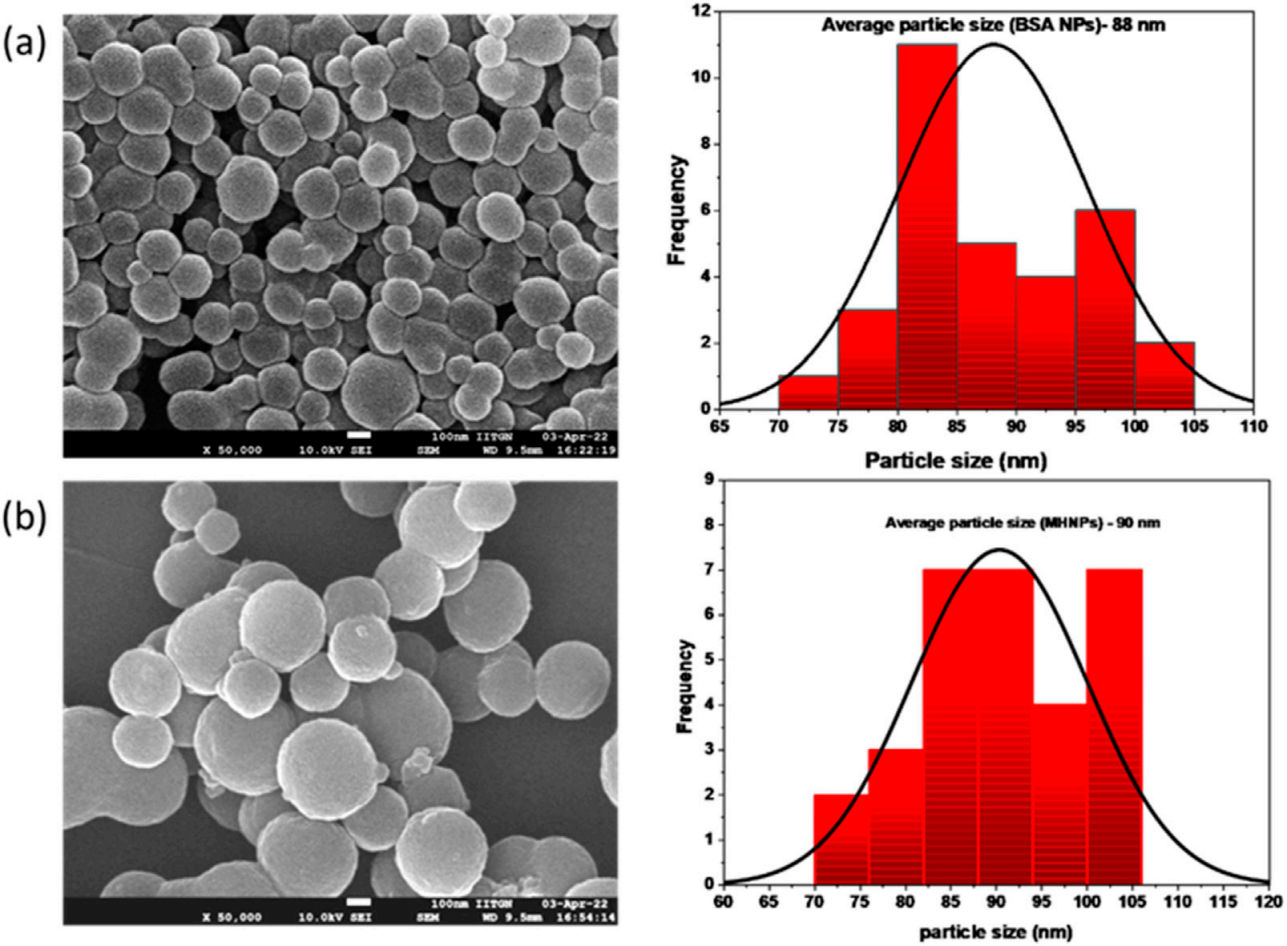
FESEM images of **(a)** BSANPs and **(b)** MHNPs.

Pictures of the nanoparticles reveal their spherical shape with an even surface and homogeneity with certain agglomeration property. Figure 3 shows the morphology of resulting BSANPs and MHNPs. TEM images (Supplementary Figure S1) further corroborated with the FE-SEM results. This significant reduction in the average size of dry NPs have confirmed the dissolvation and significantly increased the surface area.

#### 3.2.3 FTIR

**FIGURE 4:**
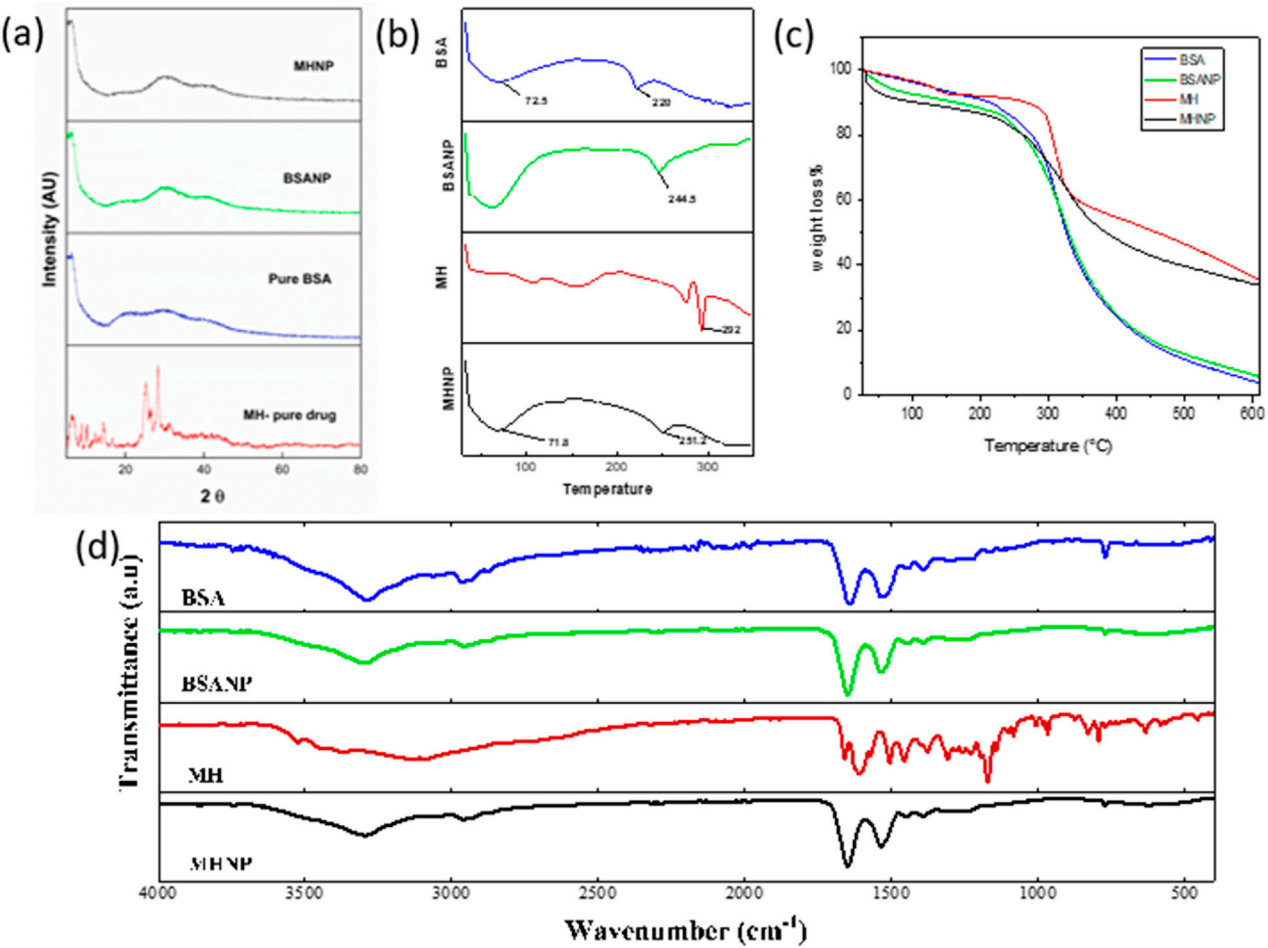
Characterization of MH-BSA-NP by **(a)** XRD **(b)** DSC **(c)** TGA **(d)** FTIR.

The observed findings in blank BSANP were equivalent to those in pure BSA. BSA did not undergo any detectable chemical changes throughout the synthesis procedure, indicating that the chemical makeup of BSA was preserved during the nanoparticle formation. In Figure 4D the FTIR spectra of pure MH revealed distinctive peaks at 3,535, 3,115, 1,613, 1,462, 1,388, and 1,307 cm^-1^, which are vibration frequencies caused by free -OH bond vibration, CH aromatic stretching, C=O, C=C, and C-OH stretching respectively. Notably, in MHNP, the O–H band around 3,360 cm^-1^ became significantly broader and less intense compared to pure MH, suggesting strong hydrogen bonding between MH and BSA (Zhang et al., 2019). The C=O stretching band of MH showed overlap and slight shifts with the amide I region of BSA, implying non-covalent interaction within the nanoparticle matrix (Ahire et al., 2025). Additionally, the reduced intensity and partial masking of MH-specific peaks in the 1,000–1,300 cm^-1^ region further support that MH is encapsulated and shielded within the BSA matrix. No new absorption bands were observed in MHNP, confirming the absence of chemical degradation or formation of new covalent bonds, and indicating that the encapsulation process preserves the chemical integrity of Morin Hydrate (Dwivedi et al., 2025).

#### 3.2.4 XRD

XRD analysis was done for determination of physical state of MHNPs. Figure 4a represents the powder XRD diffractogram of BSA, BSANP, MH and MHNPs. Sharp and imposing Bragg peaks were seen in pure MH spectra at 2θ angles 8.3, 10.4, 14.5, 16.7, 25.6, 28.4, and 31.6, indicating that MH is crystalline. In contrast, both pure BSA and BSANP displayed broad, diffuse halos lacking sharp diffraction peaks, characteristic of amorphous or semi-crystalline materials (Racz et al., 2022). The similarity in their diffraction profiles suggests that the nanoprecipitation method employed does not alter the inherent amorphous nature of BSA. The XRD pattern of the MHNP formulation showed a complete disappearance of the sharp crystalline peaks of MH, replaced by a broad halo pattern similar to BSANP. This indicates that MH underwent a phase transition from crystalline to amorphous upon encapsulation, possibly due to its molecular dispersion within the BSA matrix (Sharma et al., 2022; Shi and McHugh, 2023).

#### 3.2.5 DSC

BSA shows characteristic thermal transitions. The 220 °C peak is a sign of major structural breakdown of protein while the 75.2 °C denotes glass transition or bound water loss/protein unfolding. The shift of the degradation temperature from 220 °C (BSA) to 244.5 °C (BSANP) indicates that nanoparticle formation has improved thermal stability of BSA, likely due to cross-linking, reduced mobility of protein chains and possible encapsulation or structural reorganization. Sharp, well-defined peak suggests high crystallinity and purity of MH. Peaks observed at ∼ 71.8 °C and ∼ 251.2°C, the ∼ 71.8 °C could indicate the loss of bound water and physical changes (possible glass transition or early release of volatile components). The 251.2 °C peak Shifted lower than pure MH (292 °C) in Figure 4b, suggesting Reduced crystallinity of MH inside nanoparticles, successful encapsulation of MH within the BSA matrix. Molecular interactions between MH and BSA Amorphization of MH in nanoparticulate form which is desirable for improved solubility and bioavailability.

#### 3.2.6 TGA

Thermogravimetric analysis (TGA) curves of BSA, BSANP, free MH, and MHNP are presented in Figure 4c, and a comparative summary of key thermogravimetric parameters is provided in Table 1. All samples exhibited a minor initial weight loss below 150°C, corresponding to the evaporation of moisture and volatile components. Free MH displayed an earlier onset of major degradation (∼200°C–250°C) with a gradual weight loss pattern, reflecting its relatively low thermal stability. In contrast, the MHNP formulation exhibited a clear shift in the onset of degradation to a higher temperature range (∼250°C–350°C), accompanied by an intermediate slope of weight loss, indicating enhanced thermal protection of MH within the nanoparticle matrix. BSA and BSANP demonstrated similar sharp degradation behavior starting around 280°C–320°C, suggesting that the nanoparticle preparation process did not significantly alter the thermal properties of the BSA carrier. Furthermore, the residual mass of MHNP at ∼600°C (∼8–12%) was higher than that of free MH (∼5–7%), providing additional evidence of the protective effect of the nanoparticle structure. The observed shift in degradation behavior, improved thermal stability, and higher residual mass collectively confirm the successful encapsulation of MH into BSA nanoparticles.

### 3.3 stability studies

**FIGURE 5:**
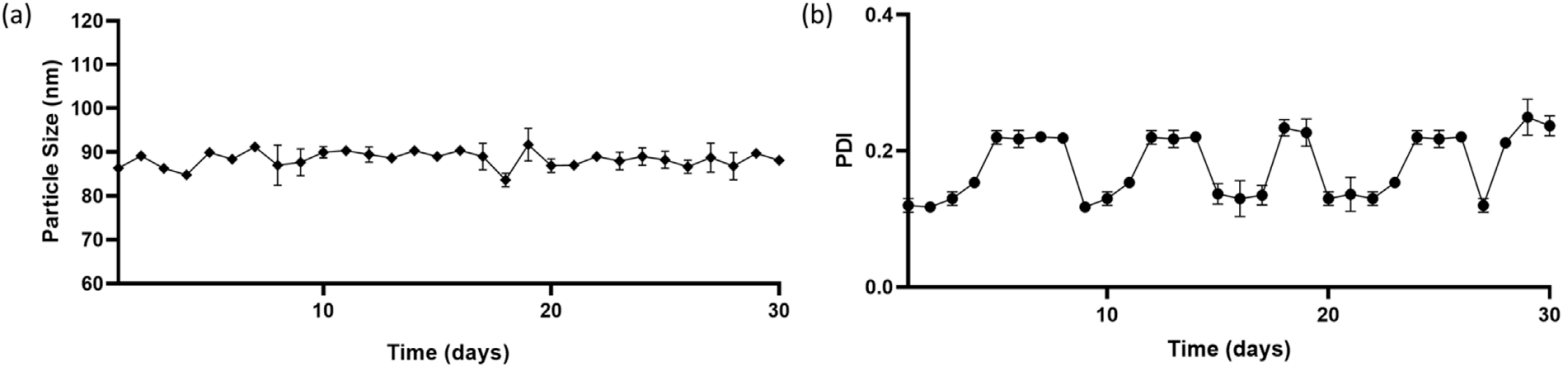
Stability of nanoparticles in terms of size and PDI over a period of 30 days **(a)** Particle size (nm) versus time (in days) **(b)** PDI (polydispersity index) versus time (in days).

Particle size and PDI of the prepared nanoparticles was monitored for a period of 30 days. Figure 5 shows the size and PDI of the NPs remained consistent with time. There was very little or non-significant variation implying greater stability to the nanoparticles. Albumin nanoparticles maintain their form and physical state even after numerous centrifugation and reconstitution cycles (Tanjung et al., 2024). This property aids to the MHNP’s utility.

### 3.4 Molecular docking

**FIGURE 6:**
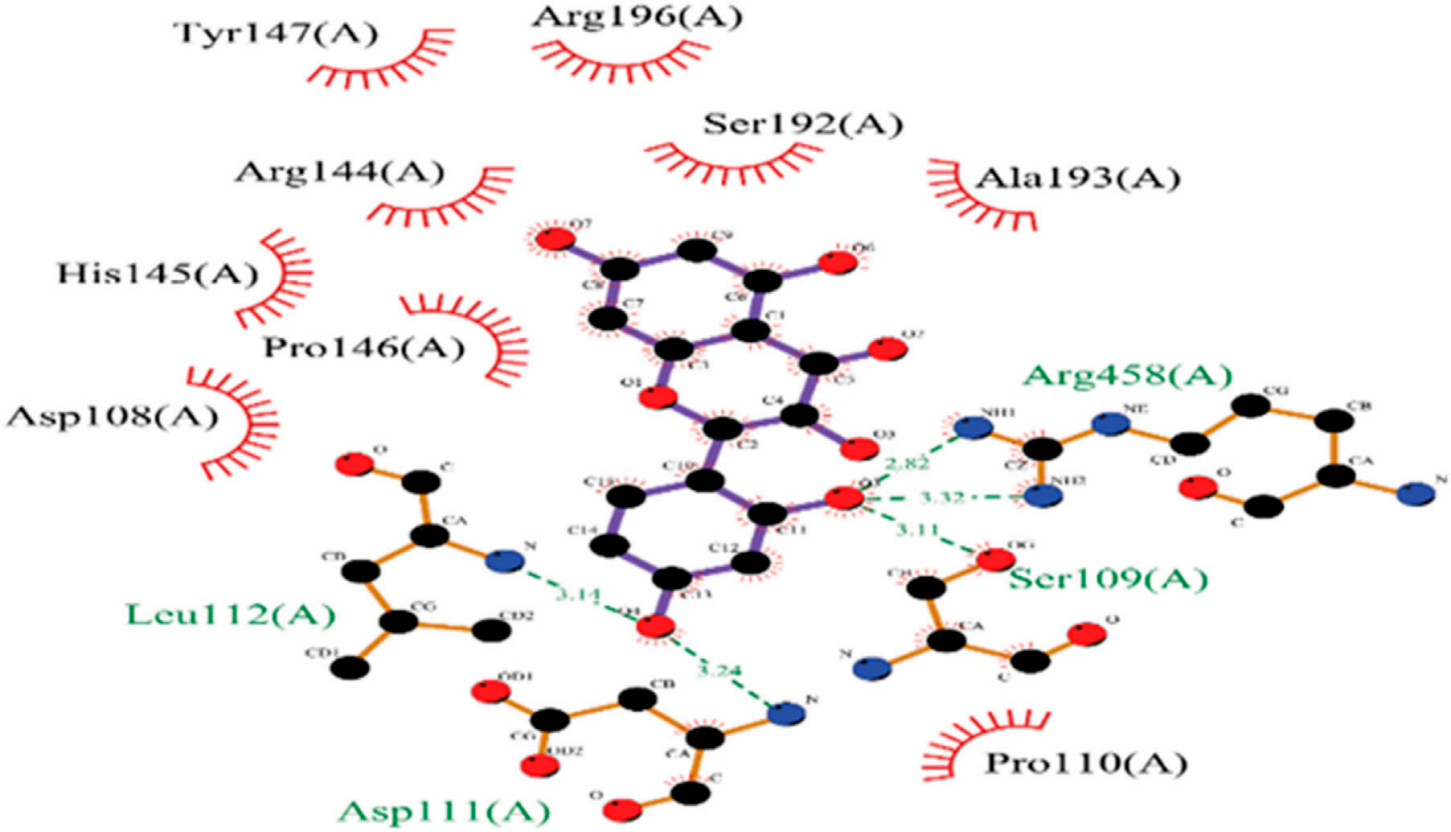
2D representation of Molecular docked structure of BSA with MH.

In the molecular docking study between BSA and MH, several key interactions were observed. MH exhibited a strong binding affinity with BSA, indicated by a binding energy of −8.4 kcal/mol. The docking analysis revealed significant interactions involving specific amino acid residues of BSA. MH formed hydrogen bonds with LEU-112(A), ASP-111(A), and ARG-196(A) residues of BSA, indicating the presence of strong attractive forces. Additionally, hydrophobic interactions, including π-sigma and π-cation interactions, were observed with PRO-110(A), ARG-144(A), and ARG-458(A) residues. These hydrophobic interactions indicated favourable stacking and van der Waals forces within the binding site. These findings suggest that the binding of MH to BSA involves a combination of hydrogen bonding and hydrophobic interactions, contributing to the stability and affinity of the complex. Overall, the molecular docking results demonstrate the potential of MH to interact with specific amino acid residues of BSA as shown in Figure 6, highlighting its potential as a promising candidate for further investigation in drug discovery or therapeutic applications.

### 3.5 MD simulation

**FIGURE 7:**
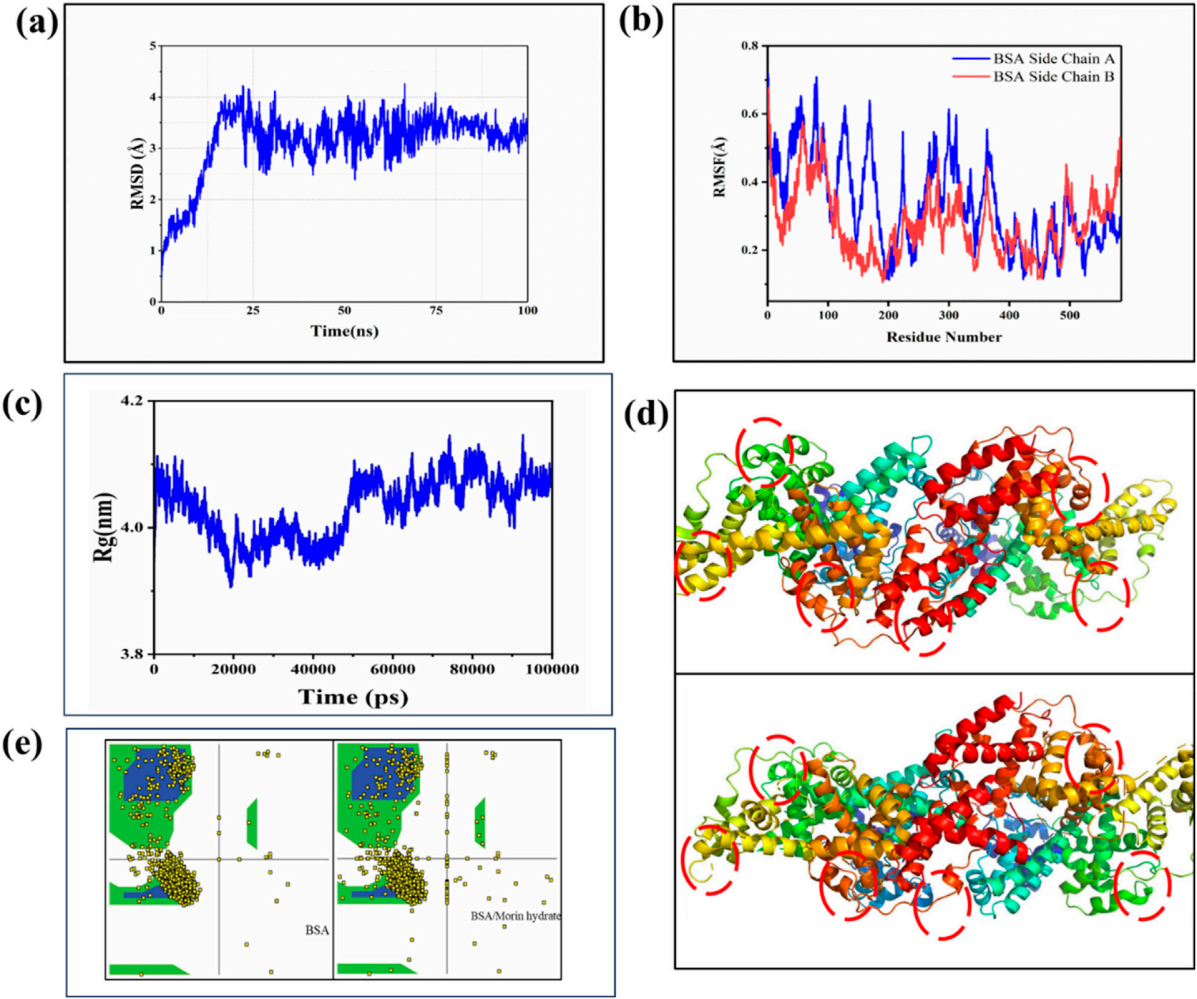
**(a)** RMSD curve plot of BSA with MH, **(B)** RMSF curve plot of BSA amino acid showing fluctuation for MH **(c)** Radius of gyration (Rg) versus time graph **(d)** 3(d) structure Conformational Changes **(e)** Ramachandran plot of BSA and BSA/MH complex.

The MD simulation of the BSA-MH complex, performed for a duration of 100 ns using the CHARMM36m force field, revealed valuable insights into the dynamics of the system. Throughout the simulation, the complex exhibited structural stability, as evidenced by a root-mean-square deviation (RMSD) value of 3.4 Å (Figure 7A). This indicates that the complex maintained its overall structure without significant deviations during the simulation period. To further analyze the flexibility of individual residues within the complex, root-mean-square fluctuation (RMSF) analysis was conducted (Figure 7B). The MD simulation of the BSA-MH complex revealed an average radius of gyration (Rg) of 4.08 Å, indicating a relatively compact and stable structure throughout the simulation (Figure 7C). The Rg values exhibited minor fluctuations around this mean, suggesting significant conformational stability and minimal unfolding events during the simulation. This observation reinforces the structural integrity of the BSA-MH complex, as the presence of MH as a ligand appears to maintain the compactness of BSA. The interaction between the ligand and the receptor did not induce any drastic changes in the overall dimensions of the complex, indicating a stable binding event. This stability is crucial for understanding the binding dynamics and potential functional implications of the BSA-MH interaction.

This analysis provides information about the fluctuation or mobility of each residue. The results of the RMSF analysis help in understanding the regions of the protein that exhibit higher flexibility or are more rigid. Taken together, the MD simulation results provide a comprehensive understanding of the BSA/MH complex. The complex demonstrated structural stability, with residues occupying energetically favorable regions. The binding between BSA and MH involved a combination of electrostatic interactions, vander Waals forces, and solvation effects, resulting in a strong binding affinity as evident from Figure 7d. Further, The Ramachandran plot analysis was performed to assess the conformational quality of the protein backbone (Figure 7e). The majority of residues in the BSA-MH complex occupied the most favored regions (A, B, L) at a percentage of 89.6%. These regions represent energetically favorable and sterically allowed regions for the backbone torsion angles. Additionally, 6.2% of residues were located in additional allowed regions (a, b, l, p), indicating some flexibility, while 2.5% of residues were found in generously allowed regions. A small portion (1.7%) of residues fell into disallowed regions, indicating potential steric clashes or unfavorable backbone conformations. The G-factor, a measure of the overall quality of the protein structure, was −0.18, suggesting a well-folded and well-behaved protein structure (Table 2). To assess the strength of binding between BSA and MH, free energy binding calculations were performed using the MM-PB(GB)SA approach. The calculated values for various energy components provide insights into the energetic contributions to the binding affinity (Table 3). The electrostatic interactions (ΔEelectrostatic) were calculated to be −51.71 ± 0.11 kcal/mol, indicating a strong attraction between oppositely charged residues in the complex. The van der Waals interactions (ΔEvdw) contributed −42.09 ± 0.94 kcal/mol, reflecting favorable hydrophobic interactions and van der Waals forces between the ligand and receptor. The solvation energies, ΔGGB and ΔGSA, were 15.30 ± 0.51 kcal/mol and −20.12 ± 0.55 kcal/mol, respectively. These values indicate the stabilization of the complex due to interactions with the surrounding solvent molecules. The enthalpy change (ΔH) was −30.25 ± 0.46 kcal/mol, indicating the overall heat energy change during the binding process. The entropy contribution (-TΔS) was 18.44 ± 0.84 kcal/mol, reflecting the changes in the system’s configurational entropy (Table 4). The resulting free energy change (ΔG) was −80.18 ± 0.19 kcal/mol, indicating a highly favorable binding affinity between BSA and MH.

**TABLE 2:** Intermolecular docking analysis of BSA amino acid with MH.

Compound	Binding energy (kcal/mol)	Amino acid	Types of bonding
BSA/MH	−8.4	PRO-110(A) (2.8)	π-sigma, Hydrophobic
		LEU-112(A) (3.1)	H-bond
		ASP-111 (A) (3.24)	H-bond
		SER-109(A) (3.1)	H-bond
		ARG-144 (A)	Hydrophobic, π – π
		ARG-196(A)	H-bond
		ARG-458 (A) (2.8,3.3)	Hydrophobic, π –cation

**TABLE 3:** Complete denoting favoured/unfavoured region and g-factor of BSA/MH complex.

	Most favored regions [A, B,L] (%)	Additional allowed regions [a,b,l,p] (%)	Generously allowed region (%)	Disallowed regions (%)	G-factor
BSA/MH	89.6	6.2	2.5	1.7	−0.18

**TABLE 4:** Free Energy binding calculation of BSA/MH complex structure.

Free energy binding (Kcal/mol)	BSA/MH
ΔEelectrostatic	−51.71 ± 0.11
ΔEvdw	−42.09 ± 0.94
ΔGGB	15.30 ± 0.51
ΔGSA	−20.12 ± 0.55
ΔH	−30.25 ± 0.46
-TΔS	18.44 ± 0.84
ΔG	−80.18 ± 0.19

These findings shed light on the molecular interactions and energetics underlying the BSA/MH complex, supporting its potential as a promising subject for further exploration in drug delivery and therapeutic applications.

### 3.6 Drug release response in simulated buffers

Drug release study was conducted to assess how improving MH solubility affects its transport through a semipermeable membrane. The most abundant plasma proteins in mammals are called serum albumins. They keep the osmotic pressure constant, which is crucial for the appropriate distribution of bodily fluids (Roy et al., 2016). Table 5 shows the pH stability of MHNPs. There is agglomeration of NPs at lower pH causing increased size and at higher pH they show a smaller size as agglomeration among particles is reduced (Supplementary Figure S2).

**TABLE 5:** Size of MHNPs in SGF and SCF.

Size of MHNPs	SGF (pH-1.2)	SCF (pH-7.4)
Day 1	235 nm	144 nm
Day 2	228 nm	192 nm
Day 3	240 nm	181 nm

**FIGURE 8:**
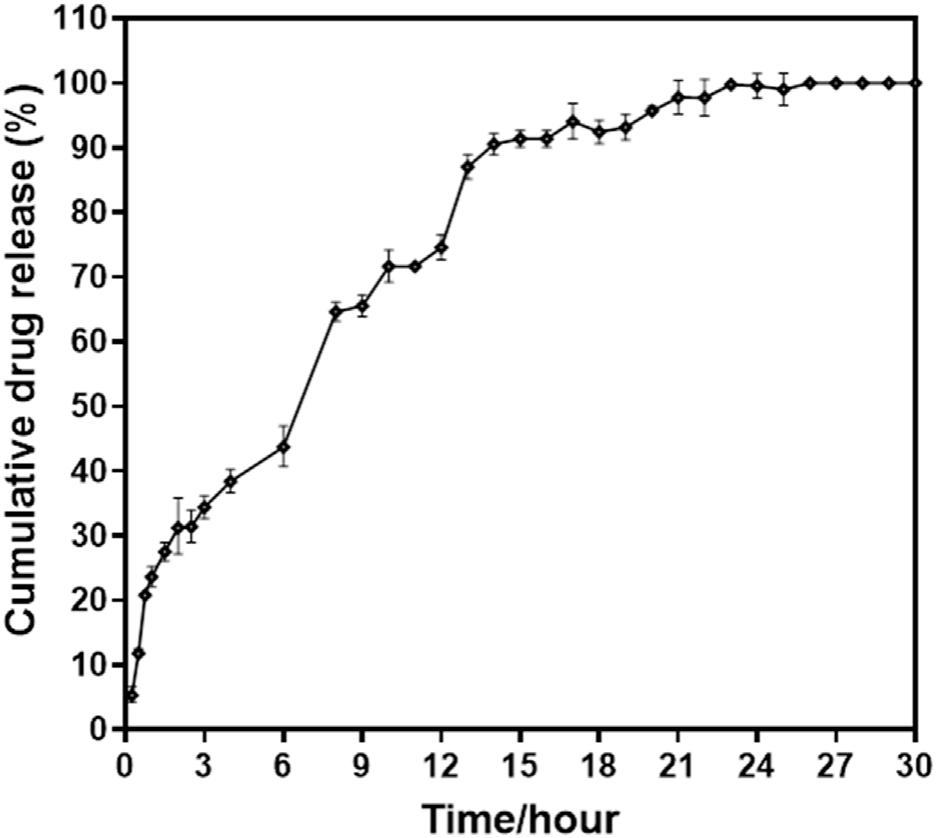
Percent cumulative drug release from MHNPs in SGF (pH- 1.2) till 3 h and in SCF (pH- 7.4) till 30 h.

Drug release from the matrix of a nanocarrier system occurs via concentration-dependent diffusion. The cumulative release of MH from NPs was detected in two release media, namely, acidic SGF and PBS, which simulate the physiological conditions of the gastrointestinal environment. Figure 8 depicts the release profile of MH in terms of cumulative percent release. It was discovered that the release of MH during the first 3 h at a strongly acidic pH of 1.2 was around 35%, and that increasing the duration from 3 h to 15 h at intestinal pH 7.4 resulted in release of 90% of total encapsulated MH and beyond till 30 h. The drug release pattern is consistent with the Higuchi model, indicating that release from the matrix of the nanoformulation. The majority of the initial release comes from the NPs’ outer surface. Drug release occurs by diffusion, which varies with concentration gradients. The medium that enters the matrix causes the particles to expand and release the drug. Thus, during the course of this study, 100% MH was released around 24 h. Additionally, readings were recorded till 30 h as shown in the graph Figure 8.

### 3.7 Antioxidant assay

**FIGURE 9:**
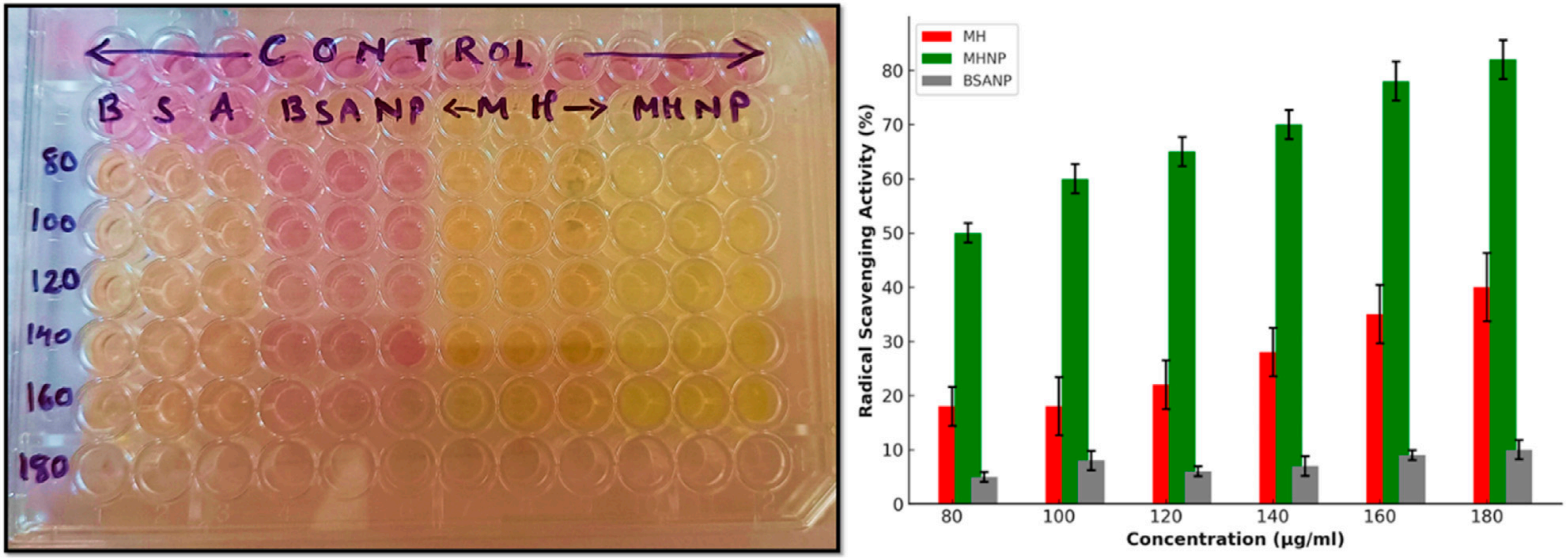
Radical scavenging activity of the experimental groups BSA, BSANP, MH and MHNPs.

Several studies report antioxidant activity is observed to accompany a significant reduction in the carcinogenesis. Moreover, flavonoids are known to alleviate chemotherapy-induced adverse effects affecting the gastrointestinal tract (Mariyappan et al., 2017). The antioxidant activity of the samples was evaluated using the DPPH assay. The radical scavenging ability of morin was assessed at various concentrations (80–180 μg/mL). The findings are shown in Figure 9. The assay revealed a concentration-dependent increase in antioxidant activity for all formulations. Among them, MHNP exhibited significantly higher scavenging efficacy compared to free MH and BSANP, reaching a maximum of approximately 78% at 180 μg/mL. This enhanced performance of MHNP could be due to the improved solubility and consistent release provided by BSA encapsulation, which likely promotes greater interaction of morin hydrate with free radicals (Brand-Williams et al., 1995; Kader, 2025). In contrast, BSANP showed minimal scavenging activity, indicating that the antioxidant potential is mainly derived from the phytochemical core rather than the protein carrier. These findings support the utility of BSA-based nanocarriers in enhancing the biofunctional performance of phytoconstituents in drug delivery systems (Kader, 2025; Sethiya et al., 2014). The DPPH assay evaluates a substance’s ability to donate hydrogen or scavenge free radicals. Flavonoids like MH have antioxidant activity due to their hydroxyl groups (-OH) (Abbas et al., 2015). The color of the test solution changes from light purple to pale, reflecting the scavenging activity of the compounds and indicating the presence of reducing compounds with antioxidant properties (Singh et al., 2013; Chakraborty et al., 2022). In the presence of DPPH, the solution’s intense purple color fades.This observation suggests that loading MH into BSANP enhances its radical-scavenging activity, likely due to improved solubility compared to pure MH.

### 3.8 Determination of IC_50_ of MH treated HCT 116 cells

**FIGURE 10:**
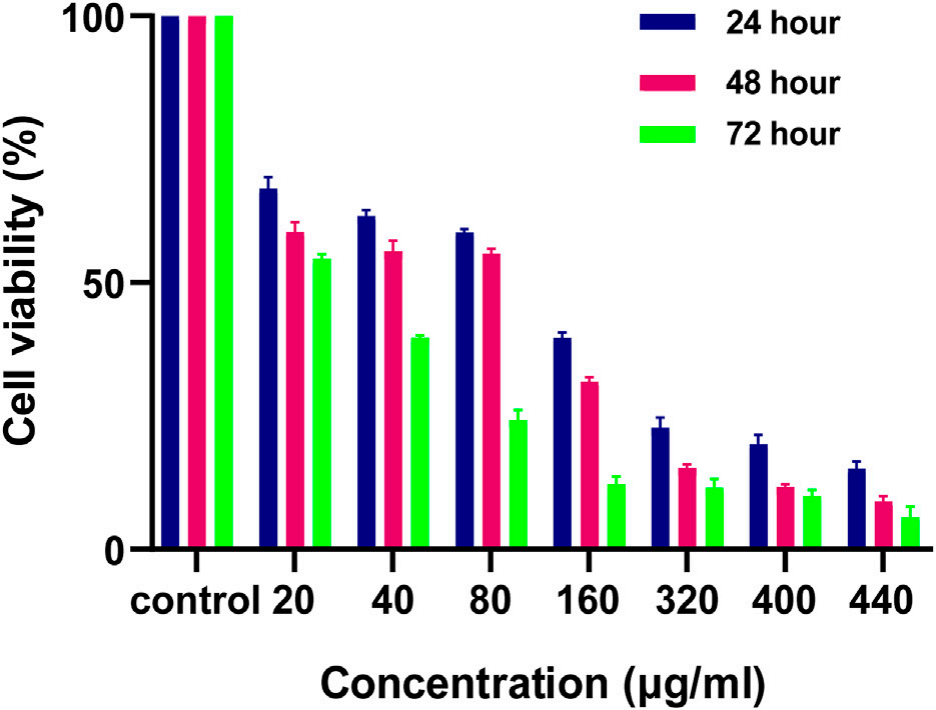
Effect of MH on HCT 116 cells after 24 h, 48 h and 72 h evaluated by using MTT Assay.

For the determination of IC_50_ of MH treated HCT 116 cells, MTT assay was done. As shown in Figure 10. 50% cell death was observed at around 80 μg/mL cytotoxic effects of MH increased in dose and time dependent manner.

### 3.9 MHNPs shows a higher cytotoxicity in dose and time dependent manner

**FIGURE 11:**
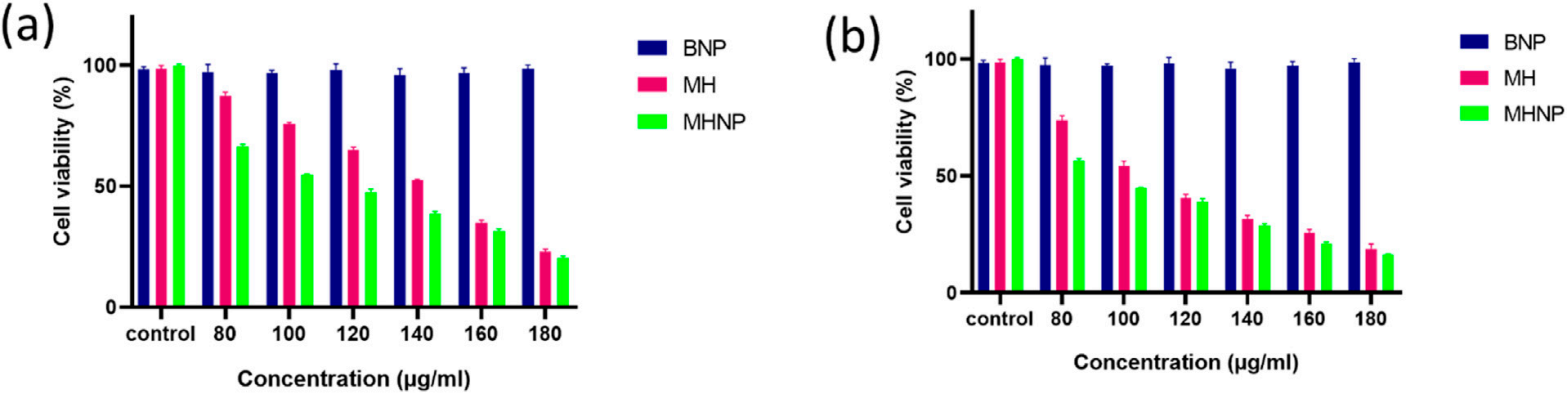
Effect of BSA NPs, MH and MHNPs on HCT 116 cells after 24 h **(a)** and 48 h **(b)** evaluated by using MTT Assay.

The assay was used to determine the cytotoxicity of MHNPs on HCT 116 cells. Figures 11A,B show that BSANPs have no toxic effects on cells, indicating that prepared nanoparticles are biocompatible. The cytotoxic effects MH and MHNPs increased in a dose and time-dependent manner with MHNPs showing higher cytotoxicity than free MH.

### 3.10 Trypan blue assay

**FIGURE 12:**
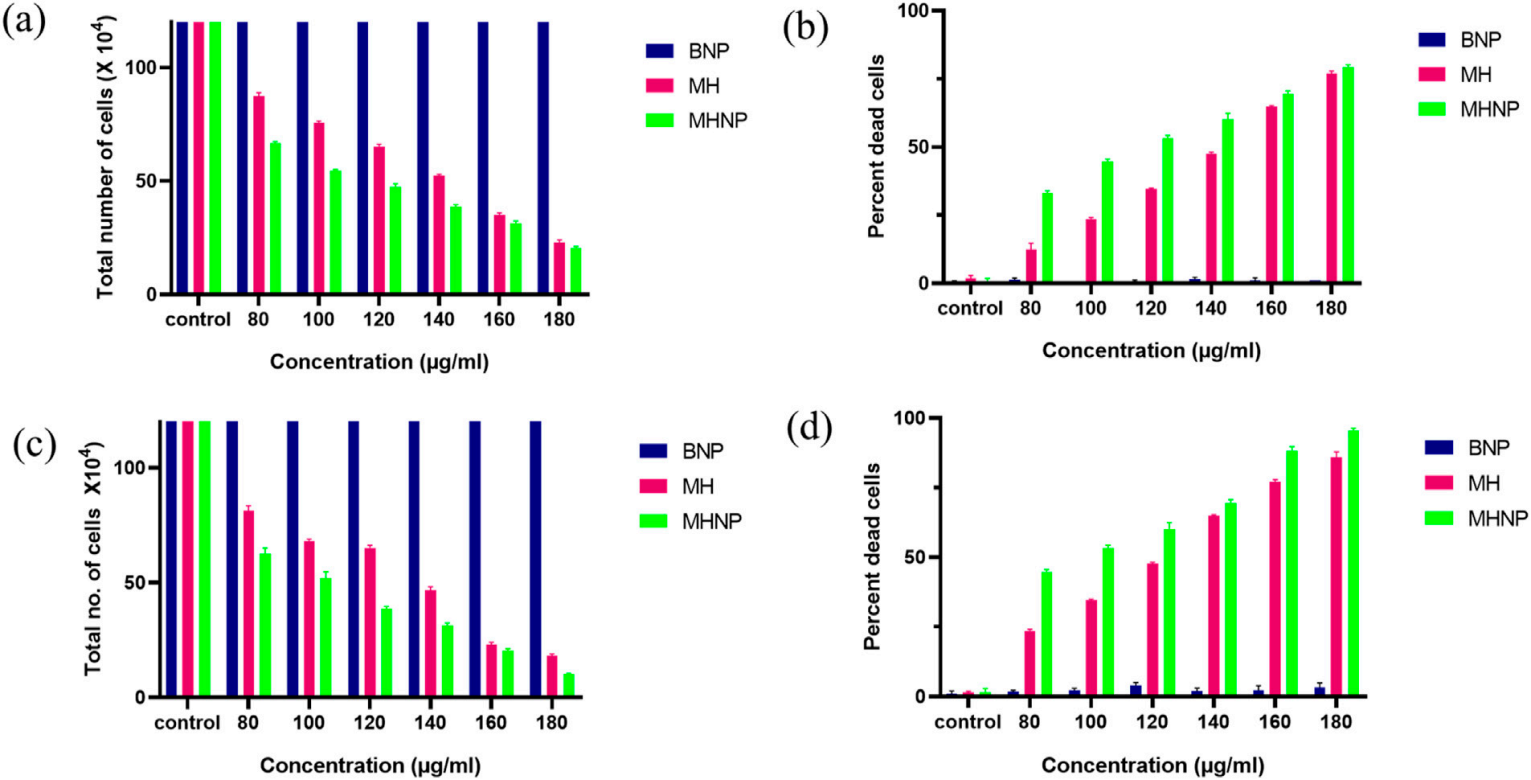
Effect of BSA NPs, MH and MHNPs on HCT 116 cells after **(a)**, **(b)** after 24 h and **(c)**, **(d)** after 48 h of treatment evaluated by using trypan blue dye exclusion assay.

For further determination of cytotoxicity, Trypan blue assay was performed. Figure 12 depicts the total number of cells versus concentration. Figure 12a shows total number of live cells versus concentration graph, where we can see 50% cell death at a lower concentration than MH alone, i.e., the IC_50_ value of drug is reduced from 140 μg/mL to 100 μg/mL 24 h after treatment and 100 to 80 μg/mL 48 h after treatment. The results corroborated with the MTT Assay data. Figure 12b shows percent dead cells versus concentration after 24 h of treatment. Similarly Figures 12c,d shows the cell viability status after 48 h of treatment.

### 3.11 Apoptosis assay

**FIGURE 13:**
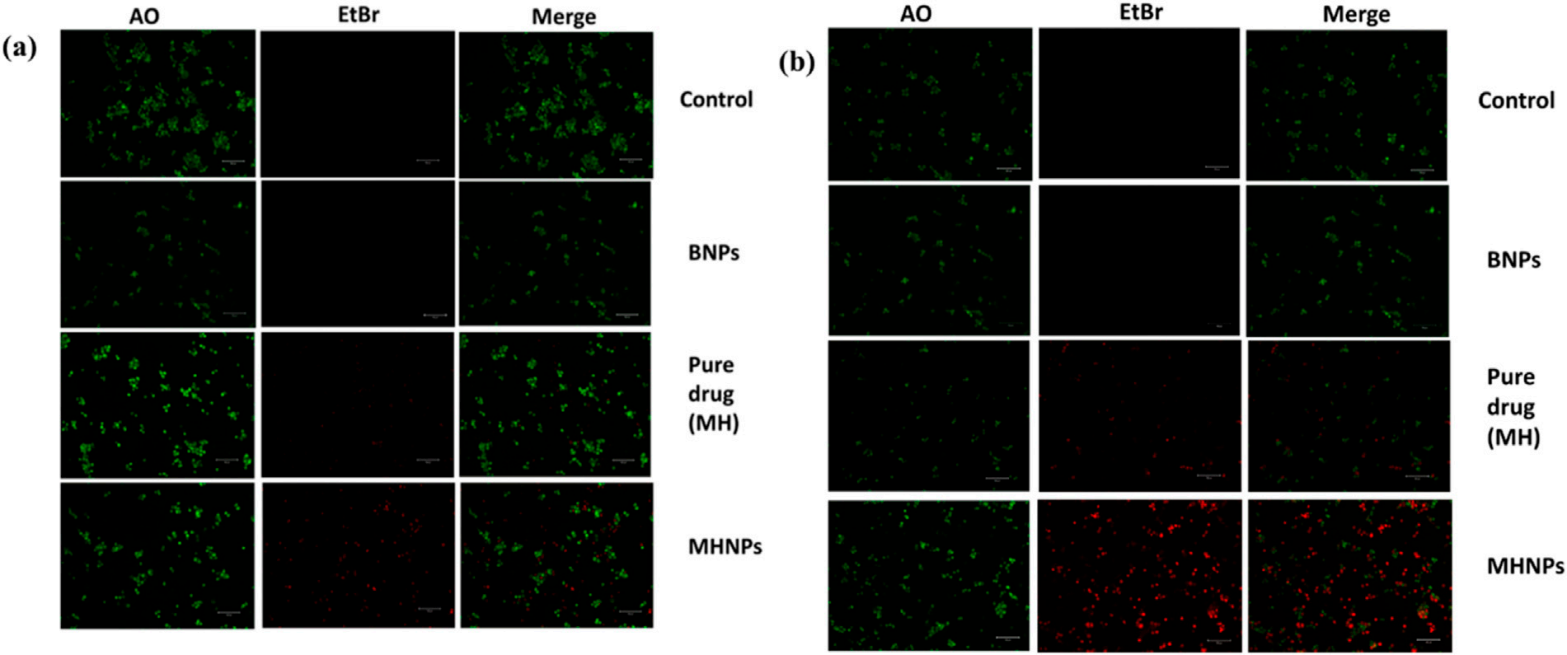
AO-EtBr staining. HCT 116 cells were treated with BNPs, MH and MHNPs at 80 μg mL^−1^ concentration for 24 h **(a)** and 48 h **(b)** and CSLM images (scale bar- 100 µM) were captured after staining with AO-EtBr dyes.

The apoptotic potential of MHNPs on colorectal cancer cells was evaluated using the AO-EtBr staining assay. Figure 13 represents CSLM images of HCT 116 cells after treatment with BNPs, Pure drug (MH) and MHNPs for 24 h and 48 h respectively. Viable cells were indicated by the untreated control cells’ mostly green fluorescence. Additionally, cells treated with BNPs flash green, indicating that the created blank nanocarrier was biocompatible with cells. Viable cells were decreased in number in the MH treated group and even higher in MHNP treated group, indicating higher efficacy of the MHNPs.

### 3.12 Colony formation assay

**FIGURE 14:**
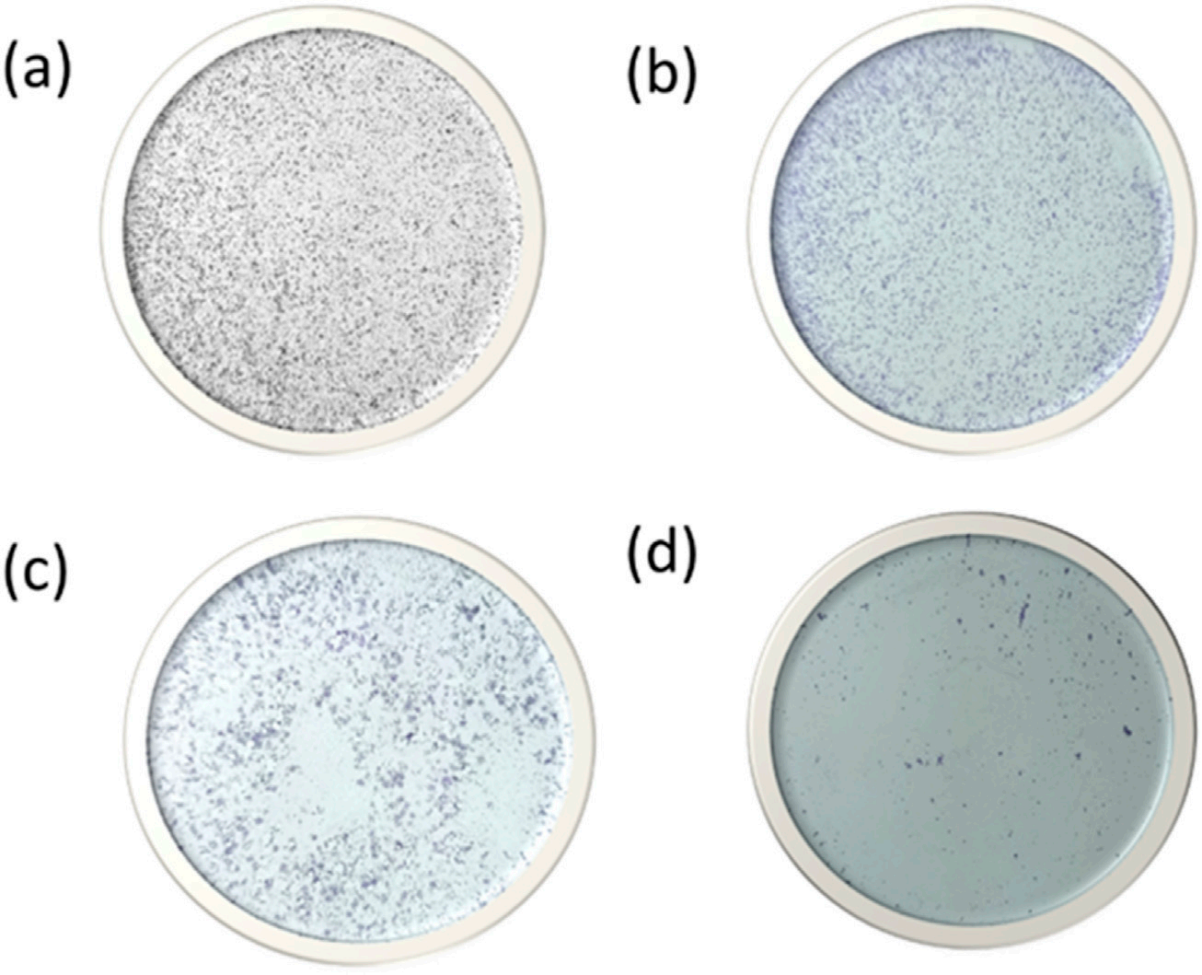
Colony formation assay after HCT 116 cells were treated with **(a)** HCT116 **(b)** BNPs, **(c)** MH and **(d)** MHNPs.

As we can see from Figure 14, Cells treated with BNPs had about identical numbers of colonies, indicating that BNPs did not significantly limit the creation of new colonies. Colony formation was markedly suppressed in colon cancer cells treated with MH and MHNPs. However, cells treated with MHNPs exhibited a greater potential for colony inhibition. The findings suggested that, in comparison to pure MH, MHNPs have more anticancer efficacy and the capacity to suppress colony development.

## 4 Discussion

This study successfully developed and characterized BSA-encapsulated Morin hydrate nanoparticles (MHNPs), with a mean size of 90 nm and an entrapment efficiency of 71.66% ± 1.5%. The encapsulation of MH within BSA nanoparticles was confirmed through various analytical techniques, including PSA, FE-SEM, TEM, FTIR, XRD, DSC, and TGA analyses. These studies demonstrated the stability, morphology, and successful encapsulation of MH within the BSA matrix, supporting its potential as an effective nanocarrier system. The PDI values of MHNPs (0.224) and BSANPs (0.244) indicate a homogeneous dispersion with acceptable polydispersity. The zeta potential values (−7.07 ± 4.51 mV for BSANPs and −11.0 ± 5.90 mV for MHNPs) suggested good stability in physiological pH conditions, minimizing aggregation. The morphological studies through FE-SEM and TEM further confirmed a uniform spherical shape with smooth surfaces, essential for controlled drug release and improved bioavailability. The FTIR spectra confirmed the presence of strong interactions between BSA and MH, with the absence of characteristic MH peaks in the MHNP complex, signifying successful encapsulation. XRD analysis indicated a transition of MH from crystalline to amorphous form upon encapsulation, enhancing solubility and bioavailability. DSC and TGA results provided insight into the thermal stability of MHNPs, demonstrating successful encapsulation of Morin Hydrate (MH) within the BSA nanoparticle matrix, as well as a significant enhancement in its thermal stability. Compared to free MH, which exhibited early onset of degradation and a continuous weight loss profile, MH-loaded nanoparticles (MHNP) demonstrated a delayed degradation onset and a more stable weight loss pattern. This shift in thermal behavior is indicative of strong interactions between MH molecules and the BSA carrier, likely through hydrophobic interactions, hydrogen bonding, or physical entrapment within the nanoparticle core. The improved thermal stability of MHNP suggests that encapsulation effectively protects MH from premature thermal degradation, which is particularly advantageous for pharmaceutical applications. Enhanced thermal stability can contribute to improved shelf life, facilitate processing steps such as spray drying or lyophilization, and ensure greater drug integrity during storage and handling. Furthermore, the protective effect of the BSA matrix may support the sustained release of MH from the nanocarrier, a feature that is highly desirable for maintaining prolonged therapeutic levels in anticancer treatment. The encapsulation strategy also enhances the potential of MH as a phytochemical-based anticancer agent, improving its physicochemical properties and bioavailability while offering a versatile nanocarrier platform for targeted drug delivery. Molecular docking studies revealed strong binding interactions between MH and BSA, having a binding energy of −8.4 kcal/mol. The interactions were stabilized through hydrogen bonding (LEU-112, ASP-111, ARG-196) and hydrophobic forces (PRO-110, ARG-144, ARG-458), ensuring effective drug loading and sustained release. MD simulation over 100 ns demonstrated the structural stability of the BSA-MH complex, as confirmed by RMSD, RMSF, and Ramachandran plot analyses. The binding free energy calculations indicated a highly favorable interaction (ΔG = −80.18 ± 0.19 kcal/mol), further reinforcing the stability of the drug-protein complex (Ying et al., 2021). The *in vitro* drug release studies of BSA-encapsulated Morin Hydrate nanoparticles (MHNP) demonstrated a significant enhancement in sustained release behavior compared to pure Morin. The release was found to be pH-responsive, with faster release in acidic (pH 5.0, SGF) conditions and a more controlled, prolonged release at neutral and colonic pH (pH 7.4, SCF). This suggests that BSA matrix protects Morin under gastric conditions and enables its gradual release in the intestinal/colonic environment, which is advantageous for site-specific anticancer delivery. The release kinetics best fitted the Higuchi and Korsmeyer-Peppas models, indicating a diffusion-driven and matrix erosion-controlled mechanism. The DPPH radical scavenging assay demonstrated that MHNPs exhibited superior antioxidant activity compared to pure MH, indicating enhanced bioactivity upon encapsulation. The improved antioxidant potential is attributed to the stable and controlled release of MH from the nanoparticle system, ensuring prolonged efficacy. This observation aligns with previous studies highlighting the role of flavonoid-based nanoformulations (Zamani et al., 2019) in mitigating oxidative stress and related pathological conditions.

## 5 Conclusion

BSA encapsulated MH nanoparticles were prepared and characterized as an efficient drug delivery system in comparison to the MH (drug) alone. The DSC analysis provided strong evidence of successful encapsulation of Morin Hydrate (MH) within the BSA nanoparticulate matrix. The disappearance of the sharp melting peak of MH at 292°C in the MHNP thermogram, replaced by a broadened endothermic transition at approximately 251.2°C, clearly indicated a reduction in crystallinity and molecular dispersion of MH in the amorphous form within the nanoparticles. This transformation is expected to significantly improve the solubility and bioavailability of MH upon administration. Furthermore, the observed enhancement in thermal stability of BSA nanoparticles (shift from 220°C in native BSA to 244.5°C in BSANP) confirmed the formation of a more stable nanoparticulate carrier system. Overall, the DSC results corroborate the effective development of a BSA-based nanoformulation capable of providing improved delivery potential for MH in pharmaceutical applications. The enhanced thermal stability of MH loaded BSA nanoparticles, as evidenced by TGA analysis, confirms effective encapsulation and underscores the promise of this nanocarrier system for achieving sustained release and improving the bioavailability of phytochemical-based anticancer therapeutics. These findings support further development of BSA nanoparticles as a versatile platform for targeted and controlled drug delivery applications. The FTIR spectral changes observed in the MHNP formulation—including O–H broadening, C=O shift, and masking of aromatic and C–O bands—provide strong evidence of successful encapsulation of MH within BSA nanoparticles via non-covalent hydrogen bonding. The XRD findings demonstrate that the encapsulation of Morin Hydrate into BSA nanoparticles results in a complete loss of crystallinity, yielding an amorphous nanocomposite that is expected to exhibit superior solubility and biological performance, critical for effective delivery in cancer therapeutics. This study highlights the successful formulation and characterization of MHNPs, with promising stability, controlled drug release, and cancer antioxidant activity. The findings suggest that BSA-based nanoparticles can serve as an efficient carrier system for MH, potentially improving its therapeutic efficacy and bioavailability. Future prospect or extension of this work could be *in vivo* studies and pharmacokinetic evaluations to further evaluate the clinical applicability of MHNPs in targeted drug delivery and therapeutic interventions. BSA nanoparticles enhance the stability and bioavailability of encapsulated drugs, allowing for targeted delivery to tumor sites (Kordkatouli et al., 2024). These nanoparticles can improve drug release profiles and reduce side effects, making them suitable for CRC therapy (Wathoni et al., 2020). While the integration of phytochemicals and nanoparticle technology shows promise for CRC treatment, challenges remain in optimizing these systems for clinical application and ensuring long-term safety and efficacy. Further research is essential to fully realize their potential in personalized medicine.

## Data Availability

The original contributions presented in the study are included in the article/Supplementary Material, further inquiries can be directed to the corresponding authors.
