# Supplementary material for: Nanoencapsulation of morin hydrate with BSA for sustained drug release in colorectal carcinoma cells: experimental and computational approach
Source: Front Drug Deliv. 2025 Aug 13;5:1623317. doi: 10.3389/fddev.2025.1623317 (PMC12360439; doi:10.3389/fddev.2025.1623317)
Supplement: Supplementary file 1 [file DataSheet1.pdf]

Nanoencapsulation of Morin hydrate with BSA for sustained drug release in colorectal carcinoma cells: Experimental and computational approach

Sanju Kumari Singh[1], Amit Kumar Srivastava[2], Sunaina Chaurasiya[3], Umesh Kumar[2], Hitesh Kulhari[3], Sunita Patel[1]

[1]School of Life Sciences, Central University of Gujarat, Gandhinagar, Gujarat, 382030, India.

[2],[3]School of Nano sciences, Central University of Gujarat, Gandhinagar, Gujarat, 382030, India.

*Correspondence:

Dr. Sunita Patel (sunitap@cug.ac.in)

Dr. Umesh Kumar (umeshkumar@cuh.ac.in)

Dr. Hitesh Kulhari (hitesh.kulhari@cug.ac.in)

Supplementary information

S1. *HR-TEM*

Surface morphology of prepared MHNPs was observed using HR-TEM (JEOL JEM2100, TEM, Tokyo Japan). A known amount of BSANPs and MHNPs was dissolved [15] 2 µL of the aqueous solution was placed onto TEM grid, dried and observed under the microscope. Particles were observed under a voltage of 80 kV. A frequency distribution curve was plotted using image J.

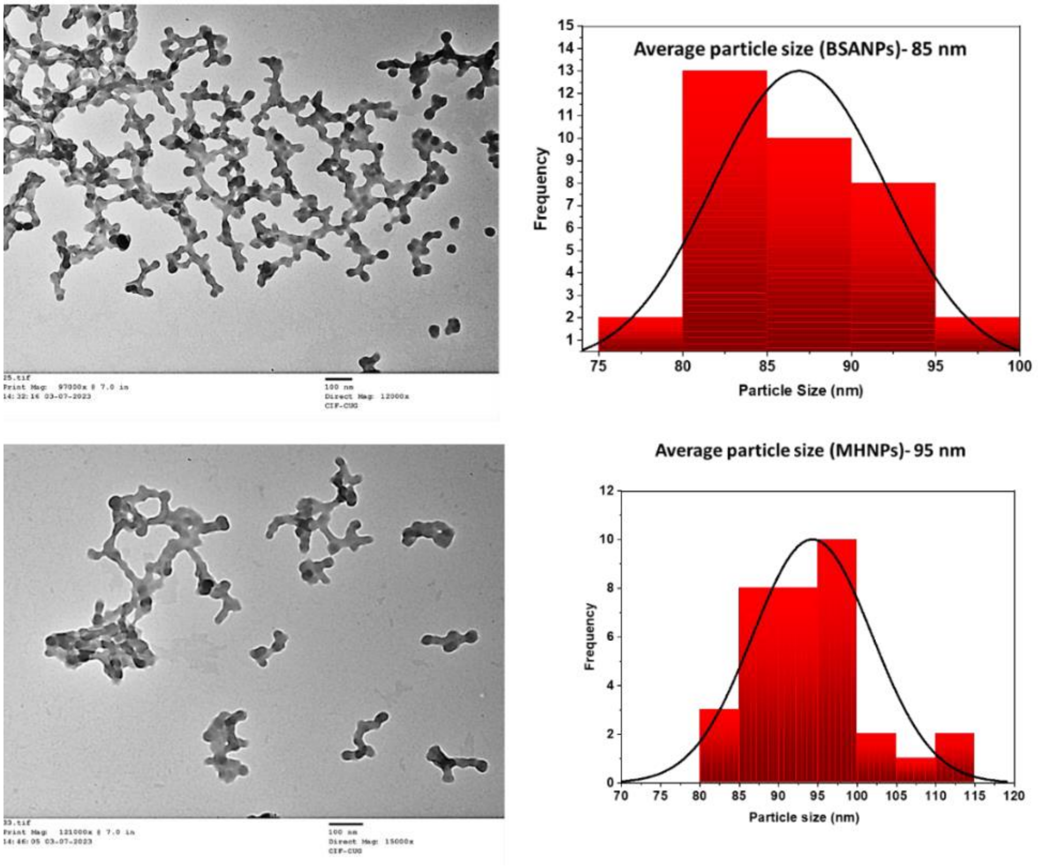

Figure S1. HR-TEM images of BSANPs and MHNPs

Size distribution graph was plotted using ImageJ

Figure S1 shows HR-TEM images which corroborated with the FE-SEM results. This significant reduction in the average size of dry NPs have greatly enhanced the surface area and confirms the dissolution process.

S2. In vitro drug release response- The in vitro drug release studies of BSA-encapsulated Morin Hydrate nanoparticles (MHNP) demonstrated a significant enhancement in sustained release behavior compared to pure Morin. The release was found to be pH-responsive, with faster release in acidic (pH 5.0, SGF) conditions and a more controlled, prolonged release at neutral and colonic pH (pH 7.4, SCF) figure S2 (a). One possible explanation for the drug's delayed release is its high entrapment efficiency within the drug-loaded nanoparticle Figure S2 (b) shows drug release pattern of MH and MHNP in SGF and SCF. Among the said groups drug surpassing 80% at 10 hour was observed in MHNP. Furthermore, there was a relatively slower release of entrapped NPs in SCF. The gradual release of the drug can be due to its high entrapment efficiency.

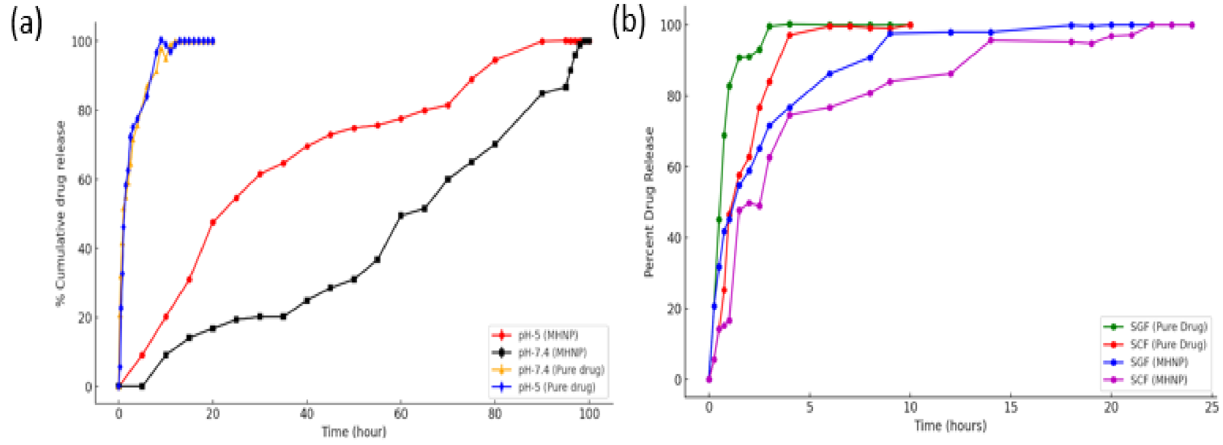

Figure S2. (a) Percent cumulative drug release at pH 5 and pH 7.4 (b) percent drug release in SCF and SGF buffers
